# ERK1/2 in immune signalling

**DOI:** 10.1042/BST20220271

**Published:** 2022-10-25

**Authors:** Richard M. Lucas, Lin Luo, Jennifer L. Stow

**Affiliations:** Institute for Molecular Bioscience (IMB) and Centre for Inflammation and Disease Research, The University of Queensland, St Lucia, QLD 4072, Australia

**Keywords:** cytokines, extracellular signal-regulated kinases, inflammation, innate immunity, receptor signalling, Toll-like receptors

## Abstract

Extracellular signal-related kinases 1 and 2 (ERK1/2) are the final components of the mitogen-activated protein kinase (MAPK) phosphorylation cascade, an integral module in a diverse array of signalling pathways for shaping cell behaviour and fate. More recently, studies have shown that ERK1/2 plays an essential role downstream of immune receptors to elicit inflammatory gene expression in response to infection and cell or tissue damage. Much of this work has studied ERK1/2 activation in Toll-like receptor (TLR) pathways, providing mechanistic insights into its recruitment, compartmentalisation and activation in cells of the innate immune system. In this review, we summarise the typical activation of ERK1/2 in growth factor receptor pathways before discussing its known roles in immune cell signalling with a focus downstream of TLRs. We examine emerging research uncovering evidence of dysfunctional ERK1/2 signalling in inflammatory diseases and discuss the potential therapeutic benefit of targeting ERK1/2 pathways in inflammation.

## Introduction

Cells utilise a range of receptors on their surface to sense and respond to external cues from the environment. Receptor tyrosine kinases (RTKs) are a widely studied receptor family which recognise growth factors, cytokines and hormones to control cell fate through differentiation, proliferation, survival, metabolism and migration [1]. Intracellular signal transduction from RTKs is propagated through the activation of cytoplasmic kinases including mitogen-activated protein kinases (MAPKs), which function as important signalling mediators to effect downstream responses. MAPKs are a highly conserved group of serine/threonine kinases which are expressed in a wide range of different cell types and play a central role in cell development and function [2]. The three conventional families of MAPKs in mammals are JUN N-terminal kinases (JNK1, JNK2 and JNK3), p38 kinases (p38-α, p38-β, p38-γ and p38-δ) and extracellular signal-regulated kinases (ERK1, ERK2 and ERK5), each of which exist as multiple isoforms and are activated in a cascade comprising orchestrated signal transduction through MAPKKK (MAP3K) phosphorylation of MAPKKs (MAP2Ks), in turn phosphorylating MAPKs for activation of downstream targets. The roles of these MAPKs in a large array of cellular pathways have been extensively reviewed [3].

**Figure 1.**
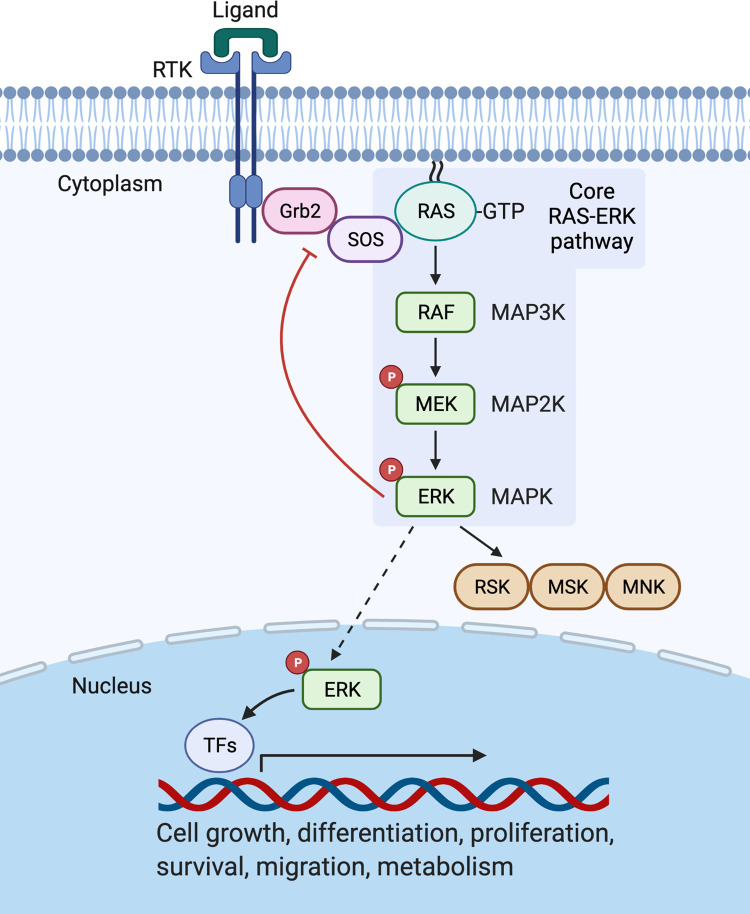
Core RAS–ERK pathway downstream of RTKs. Following ligand binding, activated RTKs recruit Grb2 via its SH2 domain. Grb2 associates with the GEF SOS which allows activation of GTP-bound RAS GTPase at the plasma membrane. As part of the core RAS–ERK pathway, RAS activation of RAF then promotes phosphorylation of MEK which phosphorylates and activates ERK. In a negative feedback loop, active ERK phosphorylates SOS to inhibit its interaction with Grb2 and prevent further RAS activation. Cytoplasmic substrates of activated ERK include RSK, MSK and MNK kinase pathways, and nuclear ERK import allows activation of its many nuclear substrates including transcription factors for gene expression in multiple cell fate pathways.

Amongst MAPK family members, ERK has attracted the greatest characterisation in the literature. The structurally related isoforms ERK1 and ERK2 were the first MAPKs to be identified through early observations of the rapid phosphorylation of two comparably sized proteins in response to growth factor stimulation [4]. Both ERK1 and ERK2 are ubiquitously expressed, with ERK2 generally expressed at slightly higher levels in most mammalian tissues, and they exhibit a level of functional redundancy in many pathways [5]. Whilst mice deficient in ERK1 are viable and show normal development, ERK2-deficient mice display severe developmental defects and embryonic lethality which is rescued by transgenic ERK1 overexpression [6]. Given their similarity, these proteins are hereon collectively referred to using the terms ERK and ERK1/2 interchangeably. In typical ERK1/2 pathways, ligand-induced activation of receptor tyrosine kinases (RTKs) at the plasma membrane activates and recruits the small GTPase RAS in its GTP-bound form which then triggers dimerisation and activation of MAP3K RAF to initiate a downstream phosphorylation cascade for sequential activation of MAP2K MEK and MAPK ERK [7] (Figure 1: Core RAS–ERK pathway). Like ERK1/2, the kinases at each level of the RAS/RAF/MEK/ERK cascade feature distinct isoforms, namely H-RAS/K-RAS/N-RAS, A-RAF/B-RAF/C-RAF and MEK1/MEK2, each encoded by independent genes which often show tissue-specific expression and provide functional diversity [8,9].

### ERK1/2 signalling from RTKs

ERK1/2 signalling has been studied extensively downstream of extracellular growth factor receptor (EGFR), an archetypal RTK with a central role in cell proliferation and association with the development of many human cancers [10]. Activated EGFR recruits the adaptor Grb2 and the guanine nucleotide exchange factor (GEF), SOS, to the plasma membrane for activation of membrane-bound RAS GTPase, thereby stimulating the MAPK phosphorylation cascade for ERK1/2 activation (Figure 1). As well as targeting of its downstream substrates, ERK-mediated phosphorylation of SOS causes its disassociation from Grb2 to create a negative feedback mechanism for curtailing RAS activation [11,12]. The recruitment of soluble kinases to specific membrane domains for receptor-mediated signalling has emerged as a critical feature dictating spatiotemporal regulation of signalling cascades. Earlier studies focused only on a general recruitment of RAS to the plasma membrane where it was assumed all receptors were activated to initiate signalling. It has since been recognised that RAS can be partitioned to endomembranes including endosomes, Golgi and endoplasmic reticulum to support RAS–ERK signalling generated by receptors at all of these sites [13]. Thus, mechanisms for the recruitment of specific kinases to subcellular membrane domains or receptors is now a key issue for consideration. To compartmentalise its activity for specific pathways, ERK1/2 is directly recruited to particular membrane domains by scaffolding proteins, which include paxillin, FHL1, GIT1 and PAK1 at focal adhesions [14], CAVIN4, ß-arrestin and MORG1 at G protein-coupled receptors (GPCRs) [15,16] and IQGAPs at the plasma membrane [17]. However, in many cases, the mechanism of ERK1/2 recruitment to these scaffolds or their precise role in ERK signalling remains to be fully established [2].

Upon its activation, ERK1/2 phosphorylates a diverse range of cytoplasmic and nuclear substrates and its repertoire of targets is further expanded through activation of MAPK-activated kinases such as ribosomal protein S6 kinase (RSK), mitogen- and stress-activated protein kinase (MSK) and MAPK-interacting kinase (MNK) pathways [18] (Figure 1). To afford access to its nuclear substrates, activated ERK1/2 is shuttled from the cytoplasm to the nucleus by diffusion through the nuclear pores, a process which is facilitated by an interaction with nucleoporins and phosphorylation of nuclear pore subunits [19,20]. A common class of ERK targets in cell proliferation and growth pathways are immediate early genes such as FOS, JUN, MYC and EGR families of transcription factors which are rapidly transcribed following mitogen stimulation of the cell [21]. In cell metabolism pathways, ERK1/2 activates key metabolic regulatory proteins such as mTOR complex 1 (mTORC1) and transcription factor HIF1α for control of protein synthesis, respiration and glucose metabolism [22,23], and plays a crucial role in cell differentiation and development through regulation of pluripotency transcription factors including OCT4, KLF2 and STAT3 [24–26].

Since its initial discovery, ERK1/2 signalling has attracted a significant weight of literature detailing its role in determining fundamental cellular behaviours [2,27,28]. Further to these diverse pathways, ERK1/2 plays an essential role in immune cells for shaping the inflammatory response to infection and cell damage [29]. In this review, we detail the known roles of ERK1/2 in immune cells with a focus on innate immune signalling downstream of Toll-like receptors (TLRs). We then highlight spatiotemporal aspects of ERK1/2 signalling in these pathways, discuss associations with inflammatory disease and finally discuss ERK1/2 signalling as a therapeutic target in these disease settings.

## ERK1/2 in immune cells

Innate immune cells provide the first line of defence against infection through the activation of germline-encoded transmembrane pattern recognition receptors (PRRs). PRRs detect molecular signatures from foreign pathogens, termed pathogen-associated molecular patterns (PAMPs), as well as endogenous damage-associated molecular patterns (DAMPs) associated with tissue damage and inflammation [30]. Activation of PRRs induces the production of pathogen-targeting molecules such as antimicrobial peptides and inducible nitric oxide synthase as well as inflammatory cytokines and chemokines for the recruitment of additional immune cells and induction of the adaptive immune response [31]. Innate immune PRRs include TLRs, NOD-like receptors (NLRs), RIG-I-like receptors (RLRs) and C-type lectin receptors (CLRs), and the activation of p38, JNK and ERK1/2 MAPK pathways by these PRRs, as part of the inflammatory response, is well described [32,33].

### ERK1/2 signalling from TLRs

**Figure 2.**
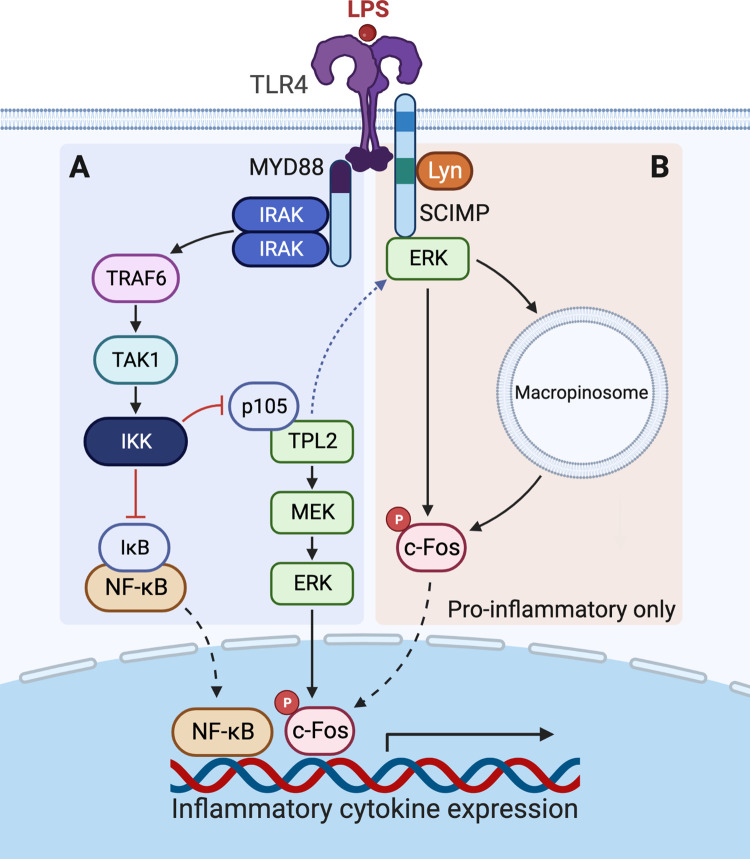
ERK activation downstream of TLR4. (**A**) LPS binding to TLR4 on the cell surface stimulates the recruitment of MYD88 through a homologous TIR domain interaction (shown in purple). Subsequent MYD88-IRAK oligomer formation activates TRAF6 and TAK1 to promote IKK-mediated degradation of IκB to allow the nuclear translocation of NF-κB for inflammatory cytokine expression. IKK also promotes proteolysis of TPL2 inhibitory protein p105 to activate the TPL2-ERK pathway for ERK phosphorylation and activation of nuclear AP-1 component c-Fos. (**B**) LPS-activated TLR4 directly interacts with transmembrane adaptor SCIMP through a TIR-non-TIR interaction. SCIMP scaffolds Lyn kinase through its proline-rich domain (PRD; shown in green) and recruits a subset of ERK to TLR4 on the cell surface and macropinosomes for phosphorylation of c-Fos which translocates to the nucleus for pro-inflammatory cytokine expression. Dashed blue arrow between pathways shows possible TPL2 activation of SCIMP-scaffolded ERK.

MAPK activation in innate immune cells such as macrophages and dendritic cells (DCs) has largely been studied downstream of TLRs, a highly conserved family of transmembrane receptors which are poised to recognise a diverse range of specific DAMPs or PAMPs both from the cell surface and within the cell from endosomal membranes [34]. Due to their exposed position, cell surface TLRs typically recognise extracellular microbial signatures, whereas endosomal TLRs detect nucleic acids from internalised pathogens as well as self-derived nucleic acids from damaged cells or tissue [35]. Stimulated TLRs recruit specific sets of cytoplasmic adaptors through their homologous toll–interleukin 1 receptor (TIR) domains which orchestrate the initiation, duration and spatial organisation of downstream signalling pathways for inflammatory gene expression, and include classical signalling adaptors MYD88, MAL, TRIF and TRAM [36]. Activation of MYD88 downstream of all TLRs, with the exception of TLR3 which instead signals solely through TRIF [37], rapidly induces the recruitment of kinases IRAK1, IRAK2 and IRAK4 to form the multiprotein ‘myddosome’ complex which triggers a signal transduction cascade to activate the inflammatory transcription factor nuclear factor κB (NF-κB) [36,38] (Figure 2A). NF-κB is a master regulator of inflammation, promoting the expression of a wide range of pro-inflammatory cytokines including IL-1ß, IL-6, IL-12p40 and TNF in response to activation of both MYD88-dependent and MYD88-independent signalling arms [39]. The E3 ubiquitin ligase TRAF6 plays a critical role in this canonical signalling cascade by activating the kinase TAK1 through adaptors TAB2/3 which then allows IκB kinase (IKK) to promote proteasomal degradation of the cytoplasmic inhibitor of NF-κB, IκB, thereby releasing NF-κB to translocate into the nucleus for modulation of gene transcription [40]. Importantly, TRAF6-activated TAK1 also induces MAPK signalling for activation of the activator protein 1 (AP-1) transcription factor complex [41] (Figure 2A). The gene expression regulatory complex AP-1 is activated in response to a broad range of external stimuli where it regulates cellular processes such as cell survival, differentiation and proliferation, and it is a key promoter of pro-inflammatory gene expression in immune cells [42]. AP-1 is formed through homodimer or heterodimer assembly of c-Fos, c-Jun and activating transcription factor (ATF) monomers and its transcriptional activity is tightly regulated though subunit abundance, composition and phosphorylation [43].

As is the case in cell growth pathways, MAPKs are intricately associated with transcription factor phosphorylation downstream of TLRs. ERK1/2 primarily phosphorylates c-Fos for inflammatory cytokine production [44,45]. c-Jun is primarily phosphorylated by JNK [46,47] which also promotes the nuclear translocation of ATF4 to form heterodimers with c-Jun for AP-1 activation [48]. p38 phosphorylates members of the myocyte-enhancer factor 2 (MEF2) family of transcription factors, MEF2A and MEF2C, downstream of activated TLRs to control anti-inflammatory IL-10 production [49] as well as stimulating expression of c-Jun, thereby influencing AP-1-mediated cytokine expression [50–52].

Whilst early studies indicated that ERK1/2 also induces NF-κB activity through direct IKK and p65 phosphorylation in TLR and IL-1 pathways [53,54], this concept has now been superseded by the premise that ERK1/2 activity instead drives gene expression changes and controls other regulators that support NF-κB activation. For instance, ERK2-mediated phosphorylation of PARP1 has been shown to control NF-κB transcriptional activation in response to TNF stimulation [55], indicating how ERK1/2 can play a role in NF-κB regulation in response to other inflammatory mediators. Ternary complex factors (TCFs) are a major direct downstream target of ERK1/2 in RAS–ERK1/2 pathways [56], and a recent study shows that ERK1/2 also controls c-Fos transcription through activation of the TCF, ELK1, to further modulate c-Fos-dependent cytokine expression following TLR stimulation [57]. As an NF-κB target gene, ELK1 regulation by IKK, therefore, provides a level of cross-talk between transcription factor signalling arms downstream of activated TLRs.

In contrast with RTK pathways which signal through RAF, immune receptors including TLRs, tumour necrosis factor receptor 1 (TNFR1) and interleukin-1 receptor (IL-1R), activate tumour progression locus 2 (TPL2), a MAP3K expressed highly in myeloid cells and fibroblasts which is involved in inflammatory responses and angiogenesis [58]. Downstream of these immune receptors and their adaptors, IKK-dependent proteolysis of p105 couples activation of NF-κB pathways to MAPK signalling by releasing TPL2 from p105 inhibition. After liberation from p105, TPL2 directly phosphorylates MEK and thereby activates downstream ERK MAPK signalling [58,59] (Figure 2A). TPL2 is also directly regulated by IKK-mediated phosphorylation which recruits the scaffold 14-3-3 to its C-terminal tail to stimulate TPL2 kinase activity [60]. In addition to controlling MEK/ERK signalling, TPL2 can also regulate the activation of p38 in macrophages [61] and neutrophils [62] as well as ERK1/2 and JNK in mouse embryonic fibroblasts [63], positioning it as an important regulator of multiple MAPKs in several cell types. Many recent studies have demonstrated that TPL2-mediated activation of ERK1/2 in immune cells can generate diverse cytokine responses, including pro-inflammatory TNF, IL-1ß, IL-6 and IL-12p40 [64–67] as well as anti-inflammatory IL-10 and IFN-ß [68,69], highlighting the many genetic targets of ERK and its substrates in inflammatory pathways.

Whilst scaffolds for targeted ERK1/2 recruitment in other receptor signalling pathways and other cells have largely been identified (see ‘*ERK signalling from RTKs*’), the mechanism by which ERK1/2 activity is compartmentalised for TLR signalling has so far remained unresolved. We previously described the atypical TLR adaptor SCIMP, a non-TIR transmembrane protein which directly interacts with TLRs and scaffolds effectors and kinases to potentiate TLR phosphorylation, pro-inflammatory signalling and cytokine output [70–72]. As an immune-specific palmitoylated transmembrane adaptor protein (pTRAP), SCIMP clusters with TLRs in lipid raft domains and tetraspanin-enriched microdomains (TEMs) on the cell surface or on endomembranes where it directly recruits the Src-family kinase Lyn for phosphorylation of both activated TLRs, and itself, to drive transient downstream MAPK signalling responses including phosphorylation of ERK1/2 [70,72]. Recently, we showed that SCIMP acts as a transmembrane scaffold for ERK1/2 in TLR pathways, rapidly recruiting ERK2 to SCIMP at TLR4 signalling sites on membrane domains, including on cell surface ruffles and macropinosomes in macrophages [73]. Using advanced live imaging techniques, it was possible to discern the recruitment and enrichment of cytoplasmic ERK2 to these dynamic membrane domains with high-spatiotemporal resolution, for the first time providing evidence for spatiotemporal partitioning of ERK during signalling in TLR pathways (Figure 2B). Biochemical analyses showed that ERK1/2 is recruited to SCIMP that is in a complex with TLR4, and this ERK1/2 primarily activates c-Fos for increased production of pro-inflammatory cytokines TNF, IL-1ß, IL-6 and IL-12p40 but not anti-inflammatory cytokines [73], in contrast with canonical TLR-activated ERK1/2 (Figure 2A) which directs a broader cytokine profile [74]. In this study and previously, SCIMP has been shown to also activate NF-κB in response to TLR stimulation [70,72,73], which may be through ERK1/2 or rather other signalling kinases, for example, MAPKs JNK and p38 also regulated by SCIMP.

Whilst it is unlikely to act in a mutually exclusive manner to TPL2-activated ERK1/2 (Figure 2B), this implies that, as a transmembrane adaptor, SCIMP scaffolds a subset of ERK1/2 to stimulated TLRs to selectively drive rapid, pro-inflammatory responses, perhaps to give spatial or temporal emphasis or indeed to control thresholding (see ‘*Homeostatic control of ERK1/2 in immune signalling*’), for pro-inflammatory ERK activation. Driving ERK1/2 signalling in TLR pathways aligns with SCIMP's promotion of ERK1/2 activation downstream of other immune receptors in DCs and B cells. As a transmembrane adaptor clustered alongside receptors within signalling microdomains, SCIMP has unique capacity to act as an immune-specific scaffold for membrane recruitment of ERK1/2 and spatiotemporal compartmentalisation of ERK1/2 signalling. With SCIMP setting a precedent as a spatiotemporal, pro-inflammatory ERK adaptor, it will be important to seek other immune adaptors, perhaps other pTRAP family members, and beyond, which can also influence innate immune activation of this critical inflammatory kinase.

### ERK1/2 in adaptive immunity

Following the innate immune signalling that issues acute inflammatory responses, ERK1/2 signalling directs the differentiation and activation of cells of the adaptive immune system, providing longer-term protection against re-infection with invading pathogens. In B cells, Raf-mediated ERK1/2 activation by B cell receptors (BCRs) involves adaptor proteins Grb2 and GRAP [75] and controls a transcription factor network to drive B cell development [76]. Using tissue-specific inactivation of ERK2 in ERK1-deficient mice to avoid embryonic lethality, ERK1/2 has been shown to be important for multiple stages of thymic development during T cell differentiation and maturation [77], and ERK2 co-ordinates B cell and CD8 T cell survival through regulation of the transcription and stability of the apoptotic BCL-2 family member, Bim [77,78]. Recently, proteomic analysis of TCR-activated CD8 T cells has shown that ERK1/2 controls many of the key transcriptional regulators of T cell differentiation and activation [79]. ERK1/2, therefore, guides multiple signalling pathways in cells of both the innate and adaptive immune systems and is emerging as a central regulator of immune cell function.

### Homeostatic control of ERK1/2 in immune signalling

The threshold of ERK1/2 activation plays an important role in determining the level and type of cellular responses. In RTK proliferation pathways, ERK1/2 signalling exhibits clear threshold effects, whereby too little ERK1/2 activation prevents proliferation, yet too much ERK1/2 activation can drive cell cycle arrest and death [80]. Similarly, ERK1/2 activation thresholds are apparent in the context of immune signalling. BCL3, an IκB protein which regulates NF-κB, also promotes TPL2 degradation to control ERK1/2 activity following TLR stimulation [81]. The stability of TPL2, therefore, determines the activity threshold of ERK1/2 required to initiate the production of inflammatory cytokines in response to TLR stimulation. The identification of SCIMP, as a pro-inflammatory ERK1/2 scaffold for TLRs [73], may also provide a level of thresholding to direct the activity of SCIMP-recruited ERK1/2 towards a pro-inflammatory output.

Another way of controlling the activation threshold of ERK1/2 signalling is through feedback inhibition. In much the same way as ERK1/2 phosphorylates upstream components in a negative feedback mechanism in RAS–ERK pathways, it is possible that ERK1/2 demonstrates a level of feedback inhibition in inflammatory pathways to constrain excessive responses. In B cells, chronic ERK1/2 activation downstream of BCR inhibits TLR-induced B cell differentiation, introducing tolerance of B cells to self-antigens and thereby providing resistance against autoimmunity [82]. However, to date, there have been no reports of direct ERK1/2 phosphorylation of upstream TLR signalling components such as TPL2, and so it remains to be seen if ERK1/2-mediated feedback inhibition or cross-talk plays a role in the homeostatic control of TLR pathways.

## Targeting ERK1/2 in inflammation

The significance of ERK1/2 in immune cell signalling is emphasised through the many ways in which internalised pathogens have evolved mechanisms to modulate ERK signalling in inflammatory pathways as a means of immune evasion. For example, *Mycobacterium tuberculosis* secretion of tyrosine phosphatase MptpB decreases ERK1/2 phosphorylation in the cytoplasm of macrophages to block the production of IL-6 and activate Akt pathways to enhance cell survival [83]. *Salmonella enterica* and *Shigella flexneri* secrete phospho-threonine lyases which target the Thr–X–Tyr activation motif of ERK1/2 and irreversibly inhibit its kinase activities through phosphate cleavage [84,85]. As well as direct targeting of ERK1/2, other bacteria such as *Yersinia spp.* and *Vibrio parahaemolyticus* secrete acetyltransferase enzymes to acetylate upstream MEK1/2, preventing its kinase activity and inhibiting ERK1/2 phosphorylation [86,87].

Given the fundamental role of MAPK signalling in the regulation of diverse cellular processes, MAPK signalling dysfunction is well established in many diseases [88]. The RAS–RAF–MEK–ERK pathway is dysregulated in approximately one-third of all human cancers through its central role in cell growth, proliferation and survival pathways [89]. In addition, a recent genome sequencing study found that activating TPL2 mutations are present in 33% of childhood spitzoid melanomas [90]. Although no cancer-associated mutations in ERK itself have been identified to date, K-RAS and B-RAF are widely recognised oncogenes in which constitutively active mutations such as K-RAS^G12D^ and B-RAF^V600E^ lead to colorectal cancer, breast cancer, leukaemia and melanoma through persistent activation of RAF–MEK–ERK and PI3K–Akt/mTOR signalling pathways [91,92]. As such, B-RAF and MEK inhibitors have shown promise as cancer therapeutics, however, the development of resistance to inhibitors through gain-of-function mutations may compromise their use in the clinic [93]. ERK1/2-specific inhibitors have been proposed as a way of overcoming resistance to these upstream targets in cancer therapy [94].

Whilst ERK1/2 signalling dysregulation in cancer has been extensively detailed in the literature, the role of ERK1/2 in inflammatory pathways underlies an emerging association with several immune-linked diseases characterised by chronic states of inflammation. Elevated ERK1/2 activity has been found in inflamed joints of patients with the autoimmune disease rheumatoid arthritis (RA) [95] and ERK1/2 inhibitors show therapeutic promise by reducing levels of RA-associated inflammatory cytokines including TNF, IL-1 and IL-6 [96]. ERK1/2 activity has also been linked with chronic lung inflammation through sustaining a cellular state of excessive NF-κB activation in bronchial epithelial cells from patients with a homozygous for the Z mutation in alpha_1_-antitrypsin, a genetic driver of early onset emphysema [97]. In rodent models of chronic alcohol-induced liver inflammation, ERK1/2 activation downstream of TLR4 promotes altered TNF expression in liver macrophages [98]. In experimental autoimmune encephalomyelitis, an animal model of human multiple sclerosis, inhibition of ERK activation using the upstream MEK inhibitor U0126 blocked IL-23 and IL-1β production in DCs to suppress Th17 and Th1 cell auto-antigen responses and reduce the severity of disease [99]. As in cancer, no inflammation-associated ERK1/2 genetic variants have been identified to date. However, genetic associations between ERK1/2 pathway components and inflammatory disease strengthens the pathogenic role of ERK1/2 signalling in these conditions. For example, a single nucleotide polymorphism (SNP) in TPL2 (rs1042058) confers increased risk of inflammatory bowel disease including Crohn's disease and ulcerative colitis [100], corresponding with enhanced ERK phosphorylation and IL-1β and IL-18 secretion in human monocyte-derived macrophages *in vitro*[101], and reduced levels of anti-inflammatory IL-10 in patient intestinal biopsies [102].

ERK signalling has also been linked to neurodegenerative conditions such as Alzheimer's disease (AD), Parkinson's disease (PD) and amyotrophic lateral sclerosis (ALS) which are characterised by aberrant protein aggregation in the central nervous system (CNS), and in which neuroinflammation is commonly a hallmark of disease [103]. ERK1/2 is emerging as a key player in the activation of microglia, the resident macrophages of the CNS, in these diseases. For example, recent *in vitro* and *in vivo* studies show that ERK1/2 promotes TLR-mediated pro-inflammatory microglial activation and disease-related gene expression in AD [104,105]. In PD models, ERK1/2 promotes leucine-rich repeat kinase 2 (LRRK2)-mediated microglia activation leading to inflammation and apoptosis as well as mounting *in vivo* inflammatory responses [106,107]. In non-immune pathways, ERK1/2 activity is associated with defects in mitochondrial morphology and function in AD [108] as well as mitochondrial biogenesis defects and dopaminergic neuronal degeneration in PD [109,110]. ERK1/2 signalling in motor neurons has been also linked to glutamate excitotoxicity and axonal transport defects associated with ALS [111,112]. Crucially, many of these pathogenic defects can be attenuated through pharmacological ERK inhibition in disease models, reflecting the broad functions of ERK signalling in neurodegenerative pathways and highlighting ERK as a potential therapeutic target in these diseases.

Further to ERK1/2 functions in immune cell signalling, its central role in many fundamental non-immune cell pathways must be considered, possibly giving rise to unintended consequences for the use of systemic MEK/ERK inhibition as anti-inflammatory therapeutics. One promising approach to avoid this global blockade of ERK pathways is through inhibition of TPL2 upstream of ERK1/2 in inflammatory pathways, thereby having a more targeted effect on ERK1/2-mediated inflammation. TPL2 itself has been linked to autoimmune inflammation in experimental autoimmune encephalomyelitis mouse models [113,114] and a genetic polymorphism in TPL2 resulting in increased expression is associated with inflammatory bowel disease [101]. Indeed, TPL2 kinase inhibitors demonstrate therapeutic potential in multiple *in vivo* models of inflammatory diseases including RA, MS, immune thrombocytopenic purpura and inflammatory bowel disease [59]. A TPL2 inhibitor (GS-4875; tilpisertib) showed promise in suppressing inflammation in primary human monocytes [115] and was taken forward to clinical trial for the treatment of ulcerative colitis, and has now been superseded by an updated molecule with greater target coverage. It is, therefore, becoming increasingly clear that targeting ERK pathways either through direct inhibition of ERK1/2 or inhibition of its upstream regulators shows therapeutic potential for the treatment of many inflammatory and autoimmune diseases. Targeting the spatiotemporal membrane recruitment of ERK1/2 through immune-specific adaptors such as SCIMP may offer another disruptive strategy. Future studies will be of great importance to progress the widespread feasibility of ERK1/2 inhibitors and interventions in the clinic.

## Perspectives

- ERK1/2 signalling plays a central role in many fundamental cell behaviours including survival, proliferation, differentiation, migration and critically shapes many aspects of the inflammatory response and fate of immune cells.
- In innate immune cells, compartmentalisation of ERK1/2 downstream of TLRs allows for spatiotemporal control of signalling and thresholding of inflammatory responses.
- ERK1/2 signalling dysregulation is linked to many human pathological conditions and emerging studies continue to reveal its impact in chronic or uncontrolled inflammation. Future studies will play an important role in advancing the therapeutic targeting of ERK1/2 pathways in inflammatory disease.

## Open Access

Open access for this article was enabled by the participation of the University of Queensland in an all-inclusive *Read & Publish* agreement with Portland Press and the Biochemical Society under a transformative agreement with CAUL.

## Abbreviations

AD: Alzheimer's disease
ALS: amyotrophic lateral sclerosis
AP-1: activator protein 1
ATF: activating transcription factor
BCR: B cell receptor
CLR: C-type lectin receptor
CNS: central nervous system
DAMP: damage-associated molecular pattern
EGFR: extracellular growth factor receptor
ERK: extracellular-related kinase
GEF: guanine exchange factor
GPCR: G protein-coupled receptor
IKK: IκB kinase
IL-1R: interleukin 1 receptor
IκB: inhibitor of NF-κB
JNK: c-Jun N-terminal kinase
MAL: MYD88 adaptor-like
MAP2K: MAPK kinase
MAP3K: MAPK kinase kinase
MAPK: mitogen-activated protein kinase
MEF: myocyte-enhancer factor
MNK: MAPK-interacting protein kinase
MSK: mitogen and stress-activated protein kinase
MYD88: myeloid differentiation primary response 88
NLR: NOD-like receptor
PAMP: pathogen-associated molecular pattern
PD: Parkinson's disease
PRR: pattern recognition receptor
pTRAP: palmitoylated transmembrane adaptor protein
RA: rheumatoid arthritis
RLR: RIG-I-like receptor
RSK: ribosomal S6 kinase
RTK: receptor tyrosine kinase
TEM: tetraspanin-enriched microdomain
TIR: Toll/interleukin-1 receptor
TLR: Toll-like receptor
TNF: tumor necrosis factor
TNFR: TNF receptor
TRAM: TRIF-related adaptor molecule
TRIF: TIR-domain-containing adapter-inducing interferon-β
